# Multidisciplinary Management for Anterior Cutaneous Nerve Entrapment Syndrome (ACNES): A Case Series Including a Long COVID-19 Patient

**DOI:** 10.7759/cureus.88703

**Published:** 2025-07-24

**Authors:** Kyeong D Lee

**Affiliations:** 1 Pediatric Surgery, General Medicine, TMG Asaka Medical Center, Saitama, JPN

**Keywords:** abdominal wall pain, acnes, anterior cutaneous nerve entrapment syndrome, chronic abdominal pain, chronic pain management, covid-19, long covid, rehabilitation, small fiber neuropathy, surgical treatment

## Abstract

Anterior cutaneous nerve entrapment syndrome (ACNES) is an underrecognized cause of chronic abdominal wall pain. While nerve block injections are typically used as conservative treatment, persistent or recurrent cases may require surgical neurectomy. This case series presents five patients, including a long COVID-19 case, who underwent surgical treatment for ACNES at our institution. We integrated clinical presentation, CT imaging, surgical findings, rehabilitation strategies, and histopathological analysis to explore a multidisciplinary treatment approach. Preoperative CT was helpful in detecting muscle atrophy or nerve visualization, which guided rehabilitation and surgical planning. Histopathology revealed chronic compressive neuropathy in all cases, even among those with mild symptoms, suggesting that pain severity alone should not dictate surgical indications. Rehabilitation was effective in reducing muscle contraction and localizing symptoms, contributing to postoperative success and recurrence prevention. Our findings support the importance of individualized, proactive management that combines imaging, surgery, and rehabilitation, and highlight the potential for COVID-19-related neuropathy to contribute to ACNES.

## Introduction

Anterior cutaneous nerve entrapment syndrome (ACNES) is a condition where the anterior cutaneous branch of the intercostal nerve is entrapped within the anterior sheath of the rectus abdominis muscle, causing localized abdominal pain [1,2]. This syndrome is significant in the differential diagnosis of abdominal pain, often being misdiagnosed as gastrointestinal or urological disorders [3]. ACNES is typically managed through conservative treatments such as nerve block injections and physiotherapy, or surgical interventions like neurectomy [4-7]. However, an optimal treatment strategy has yet to be established. In the context of the COVID-19 pandemic, various chronic pain syndromes have been reported as part of the post-acute sequelae of SARS-CoV-2 infection (commonly referred to as "long COVID") [8-12]. While musculoskeletal and neuropathic pain have been widely described, a direct association between COVID-19 and ACNES has not been previously reported. The objective of this study is to evaluate the efficacy of surgical treatment in ACNES patients and explore the potential of integrating multidisciplinary treatment approaches, including a case potentially related to post-viral inflammatory mechanisms.

## Case presentation

Case one

An eight-year-old boy presented with right inguinal pain and was referred from orthopedic surgery under suspicion of inguinal hernia. At the initial visit, Carnett's sign was observed above the pubis in the right lower abdomen, and ACNES caused by the right ilioinguinal nerve was diagnosed. The patient opted out of conservative treatment and underwent surgical exploration of the inguinal canal with subsequent neurotomy of the ilioinguinal nerve. The postoperative course was favorable, with no recurrence of pain.

Case two

**Figure 1:**
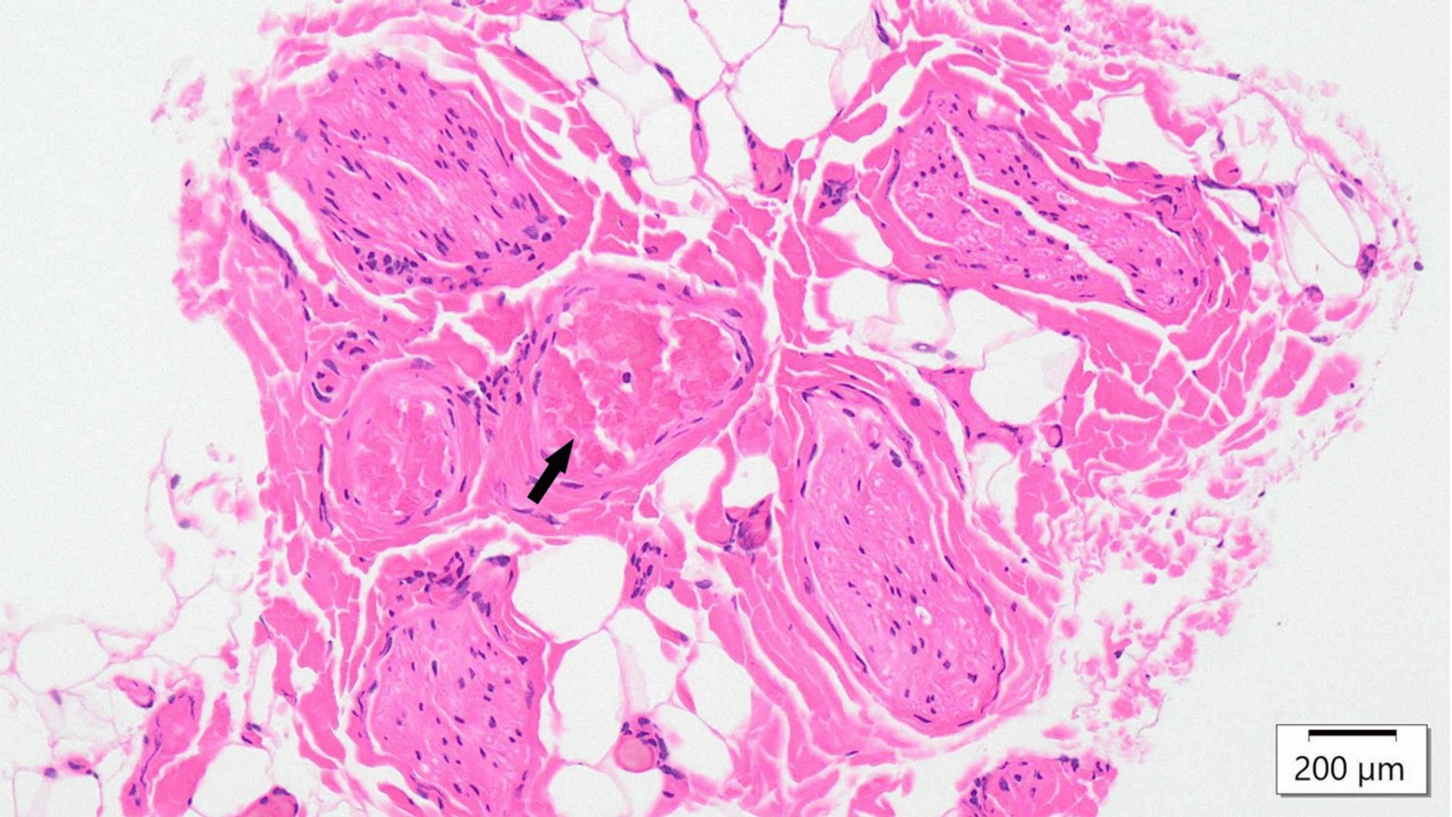
Histopathological image of the excised nerve in case two Histological image from case two showing cutaneous nerve bundles surrounded by dense perineural fibrosis and central vascular thrombus formation (arrow) (Hematoxylin and Eosin stain, scale bar = 200 μm). These findings suggest a chronic vascular compromise contributing to the nerve entrapment pathology.

A 12-year-old boy had been experiencing intermittent severe abdominal pain for a year and had visited multiple high-level medical institutions without a definitive diagnosis. He was diagnosed with ACNES of the left rectus abdominis at a previous hospital and received nerve blocks, but the pain recurred. Upon visiting our institution, severe contraction of the rectus abdominis caused by multiple offending cutaneous nerves was noted. Rehabilitation was initiated to relieve the contraction, and some cutaneous nerves were excised. Notably, before the rehabilitation, it was difficult to identify the most responsible nerve due to diffuse pain; however, the process of preoperative rehabilitation helped to clearly localize the most painful site. Histopathological findings revealed thrombosis within the vascular bundle (Figure 1). Postoperatively, the pain improved, and rehabilitation was continued for recurrence prevention.

Case three

**Figure 2:**
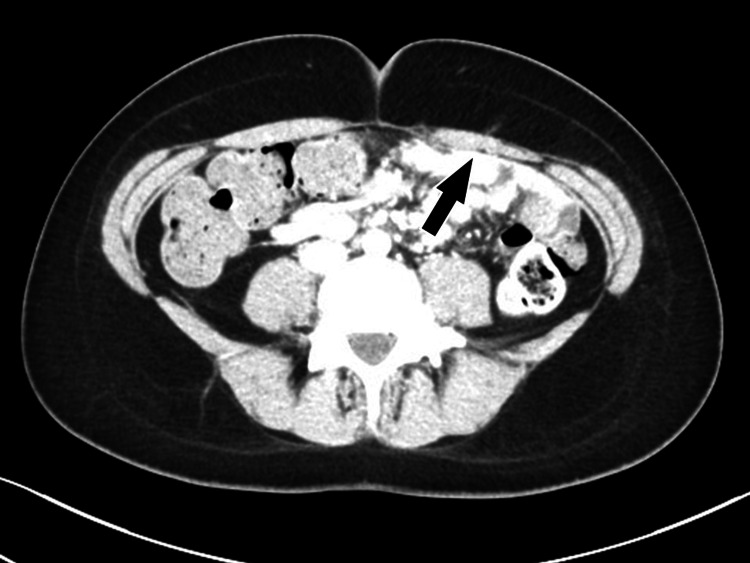
Preoperative CT image demonstrating rectus abdominis atrophy in case three Axial computed tomography (CT) scan of the abdomen in case three showing focal atrophy of the left rectus abdominis muscle (black arrow). An anterior cutaneous nerve can also be visualized extending anteriorly from the fascia, supporting the diagnosis of anterior cutaneous nerve entrapment syndrome with radiological correlation of nerve involvement.

**Figure 3:**
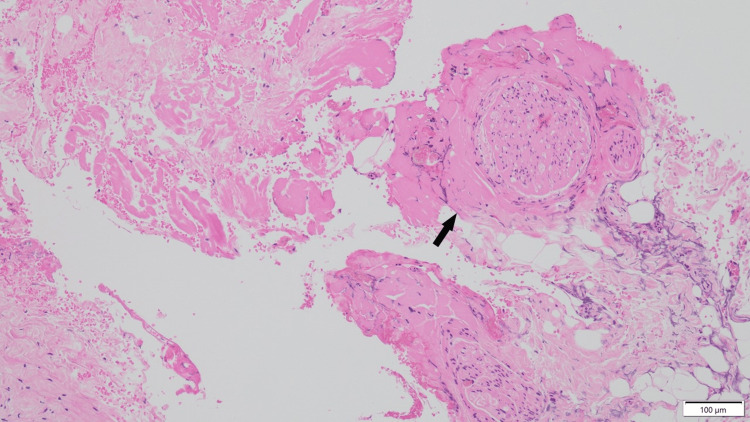
Histopathological image of the excised nerve in case three Dense perineural and endoneurial fibrosis is observed around multiple nerve fascicles (arrow), consistent with chronic compression neuropathy. The concentric collagen deposition suggest long-standing entrapment (Hematoxylin and Eosin stain, scale bar = 100 μm).

A 22-year-old woman had previously received nerve block injections and one neurectomy for ACNES at multiple medical institutions. Persistent pain led her to seek further excision of cutaneous nerves at our institution. The rectus abdominis was relaxed and atrophied, and preoperative rehabilitation was ineffective, necessitating another neurectomy (Figure 2). Histopathological findings revealed collagen fiber adhesion around the neurovascular bundle (Figure 3). Postoperatively, the pain disappeared, and rehabilitation was continued for recurrence prevention.

Case four

**Figure 4:**
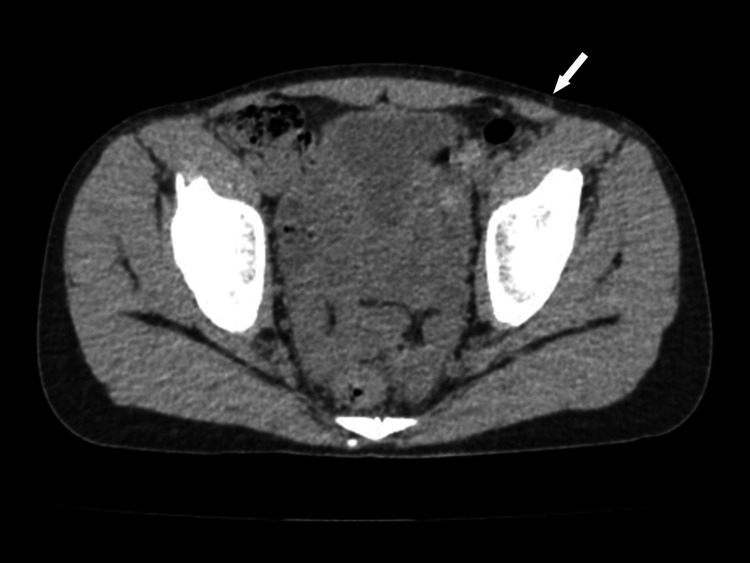
Preoperative CT image in case four showing subtle nerve-like structure at the pain site Axial CT image from case four showing no overt abnormalities in the left rectus abdominis muscle. However, a faint linear structure possibly representing the anterior cutaneous nerve can be seen at the site corresponding to the patient's reported pain (white arrow). Although subtle, this finding supports the clinical suspicion of anterior cutaneous nerve entrapment syndrome.

A 12-year-old boy presented with a six-month history of left lower abdominal pain. He had previously been diagnosed with ACNES at a local pain clinic and received nerve block injections, which provided only temporary relief. During outpatient visits, he did not exhibit pain upon examination, nor did he describe the episodes as severe when they occurred. However, he reported considerable distress and fear due to the unpredictable and sudden nature of the pain, which motivated his decision to undergo surgery. The left Th10 anterior cutaneous nerve was identified as the source of pain. Preoperative CT imaging did not reveal definitive abnormalities in the rectus abdominis muscle, although a subtle structure possibly corresponding to the cutaneous nerve was faintly visible in the area consistent with the patient's reported pain (Figure 4).

**Figure 5:**
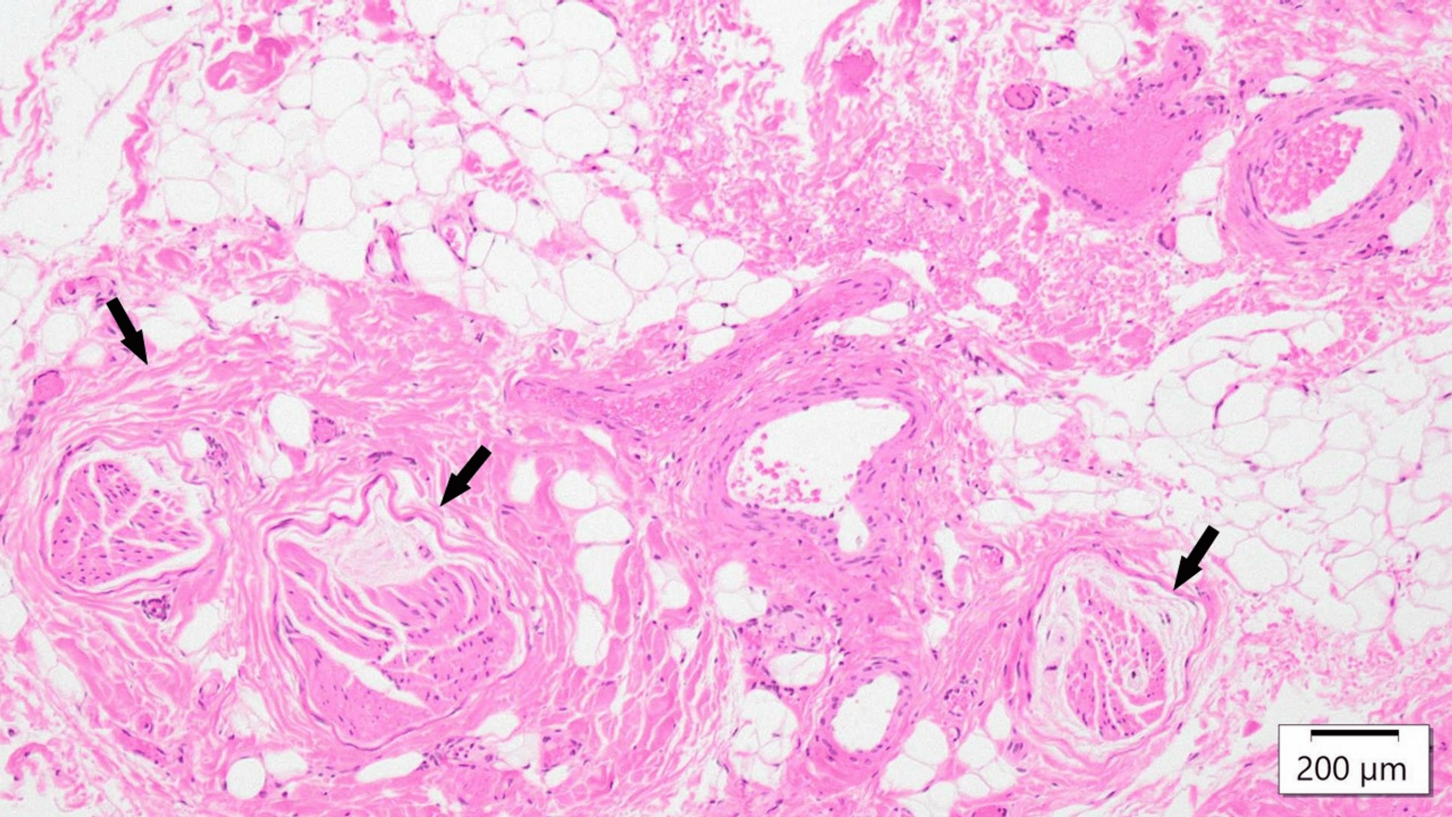
Histopathological image of the excised nerve in case four Multiple nerve fascicles show perineural fibrosis and wavy configuration of the axons, consistent with chronic nerve entrapment. Black arrows highlight distorted nerve fiber architecture with irregular alignment and stromal condensation (Hematoxylin and Eosin stain, scale bar = 200 μm).

A neurectomy was performed, and the pain resolved immediately after surgery. Histopathological examination revealed features of chronic compressive neuropathy, including perineural and endoneurial fibrosis, a wavy appearance of nerve fibers, and onion bulb-like structures - findings consistent with chronic nerve entrapment (Figure 5). The patient did not require postoperative rehabilitation and has remained symptom-free.

Case five

**Figure 6:**
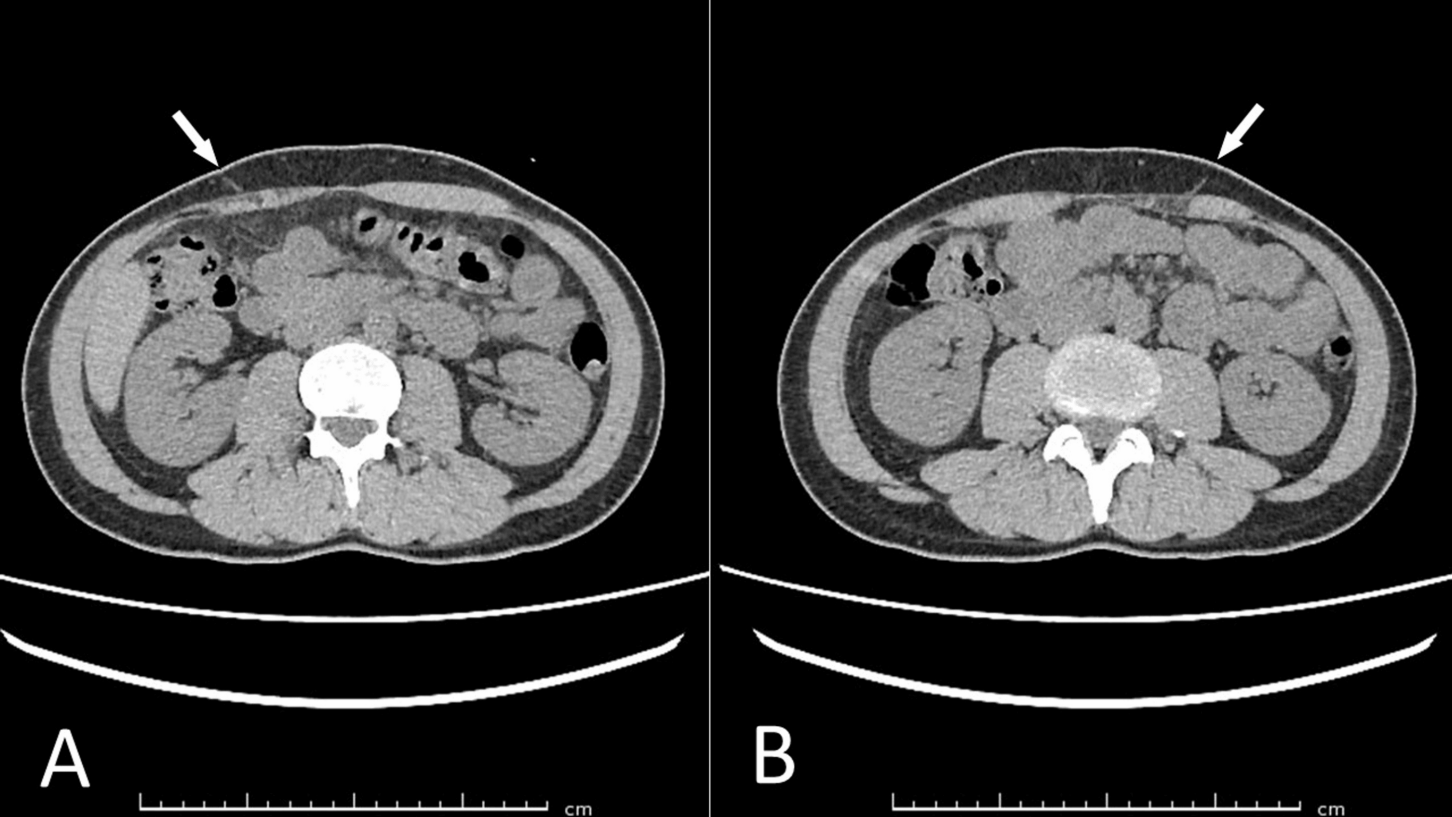
Preoperative CT images of the abdominal wall in case five Axial CT image of the abdominal wall. (A) The white arrow indicates a visible anterior cutaneous nerve bundle. Note the localized thinning of the right rectus abdominis and thickening of the anterior sheath fascia, consistent with chronic mechanical compression. (B) The white arrow points to a similarly visible cutaneous nerve bundle. Although the patient reported no pain on this side, focal atrophy of the rectus abdominis suggests subclinical muscle degeneration and potential nerve involvement.

A 34-year-old male general medicine physician presented with electric shock-like pain at rest in the right lower thoracic region. He developed symptoms consistent with ACNES involving the right Th8 intercostal nerve after recovering from COVID-19 two years prior. According to the WHO clinical progression scale, his initial COVID-19 illness would be classified as mild to moderate, characterized by high fever and upper respiratory symptoms without hypoxia, lasting four days. Mild abdominal discomfort, initially attributed to excessive coughing, persisted thereafter. Over the course of a year, the symptoms gradually worsened, and by one year post-infection, the patient began to clearly recognize the progression of pain. Six months before presentation, he developed electric shock-like pain in the same region, which became persistent and debilitating. CT imaging revealed a visible right Th9 anterior cutaneous nerve, thinning of the rectus abdominis in the corresponding dermatome, and thickening of the anterior sheath fascia (Figure 6A).

**Figure 7:**
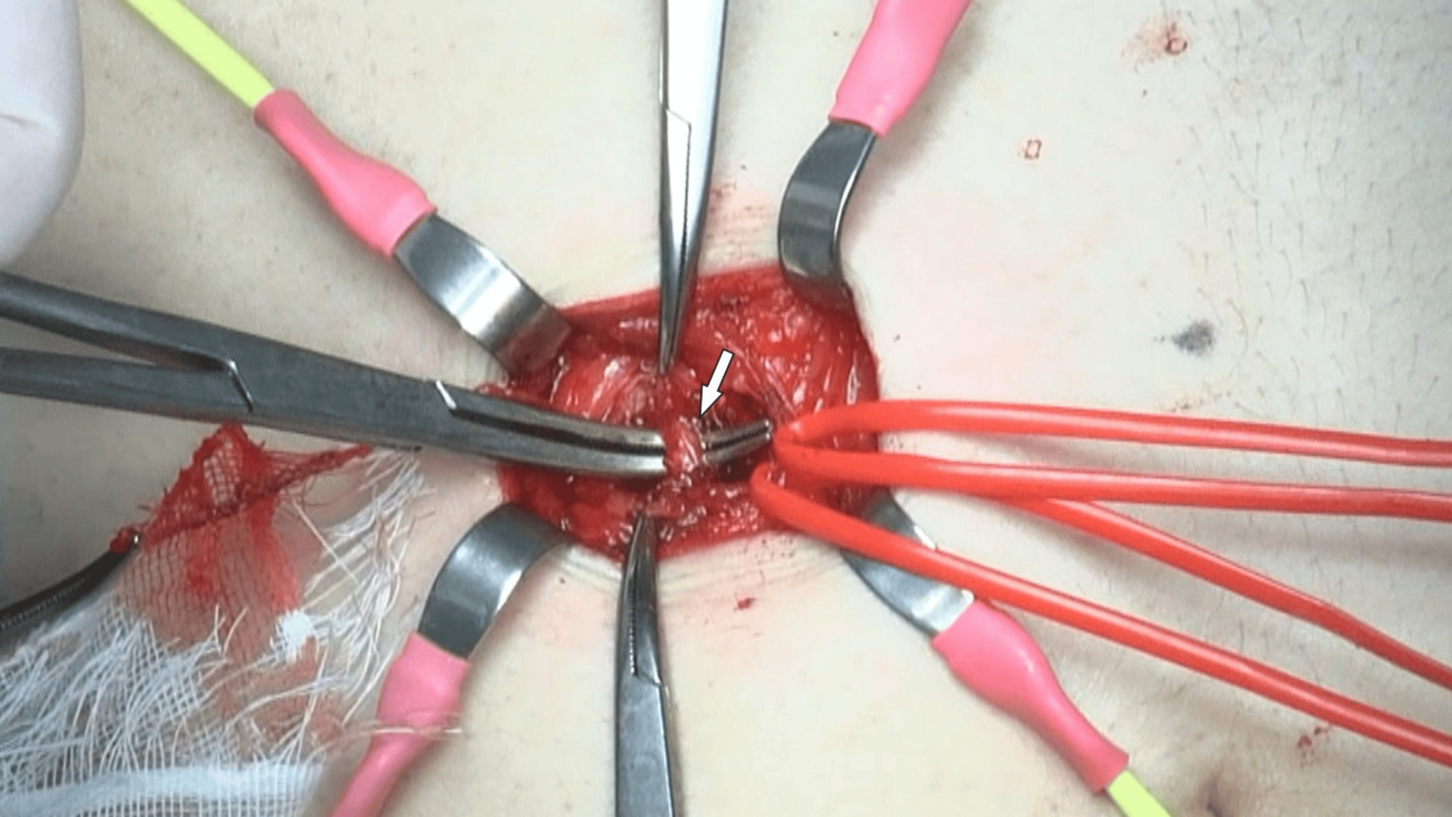
Intraoperative view of the anterior cutaneous nerve and associated vasculature in case five A thick blood vessel (white arrow) is shown in close contact with the anterior cutaneous nerve during dissection of the right Th9 intercostal region. The vessel was ligated and transected to relieve suspected vascular compression contributing to the neuralgic symptoms.

A neurectomy of both the right Th8 and Th9 anterior cutaneous nerves was performed. During dissection around the Th9 nerve, a thick blood vessel closely adherent to the nerve was identified, ligated, and transected (Figure 7).

**Figure 8:**
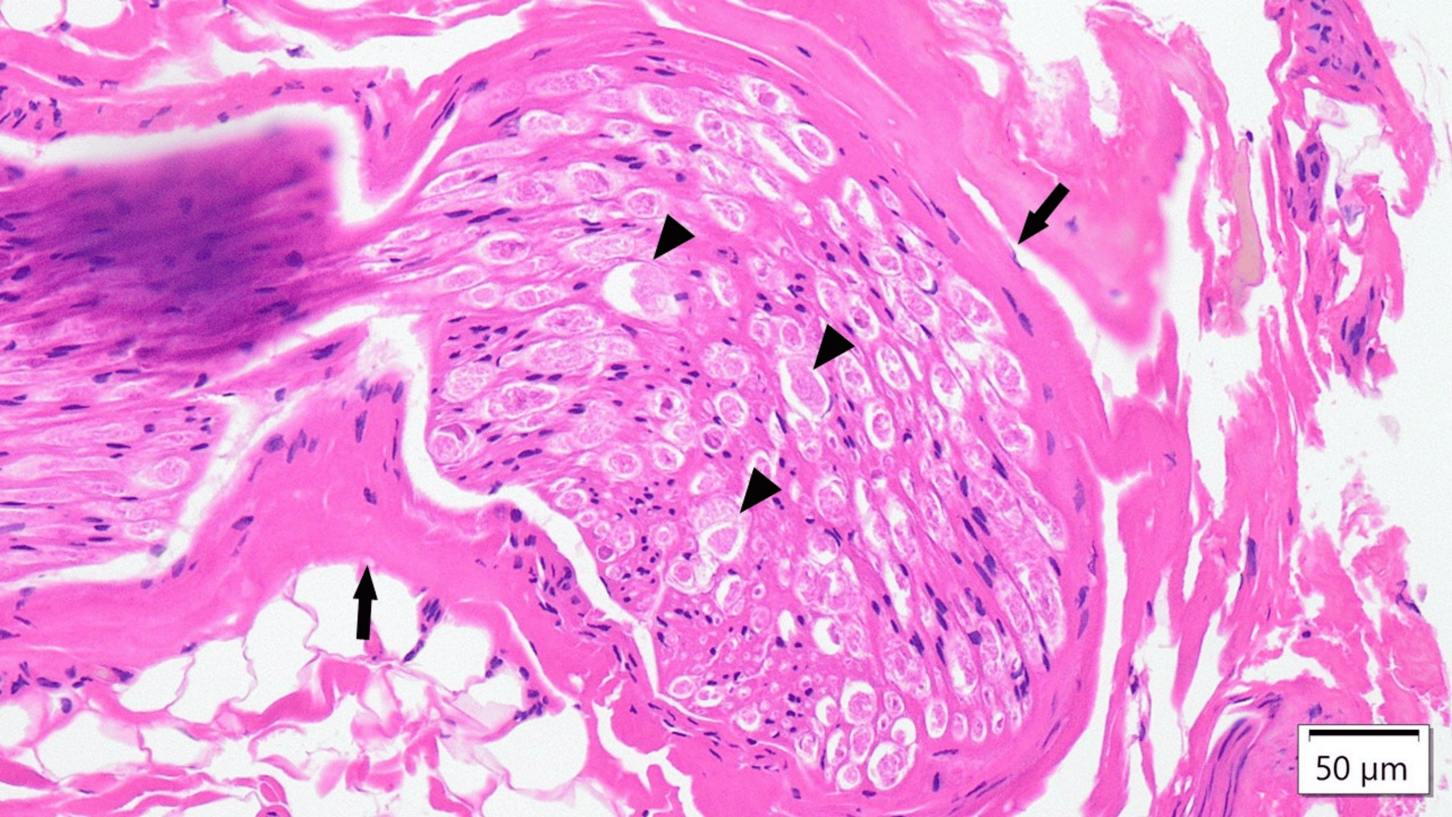
Histopathological image of the excised Th9 nerve in case five A dense cluster of small, rounded nerve fibers with surrounding myxoid matrix is observed. Arrowheads indicate vacuolated or degenerated axons, suggesting chronic axonal injury. Black arrows highlight fibrotic changes and stromal thickening around the nerve bundle. These findings are consistent with chronic compressive neuropathy (Hematoxylin and Eosin stain, scale bar = 50 μm).

The electric shock-like pain resolved immediately after surgery. Histopathological analysis of the excised Th9 nerve revealed small, clustered nerve fibers with vacuolated axons and surrounding fibrotic changes, consistent with chronic compressive neuropathy (Figure 8). Preoperative CT also revealed focal thinning of the left rectus abdominis and a visible cutaneous nerve on that side (Figure 6B). Therefore, rehabilitation was continued for recurrence prevention. 

## Discussion

This case series highlights several important aspects in the diagnosis and treatment of ACNES. The condition was observed across a wide age range, including children and adults, and was often diagnosed based on localized abdominal pain and a positive Carnett's sign. In all cases, neurectomy proved to be effective, especially when conservative treatments such as nerve blocks provided only temporary relief or when the pain significantly impaired the patient's quality of life (Table 1).

**Table 1:** Summary of five ACNES cases undergoing surgical treatment ACNES - anterior cutaneous nerve entrapment syndrome

Case	Age / Sex	Duration of symptoms	Pain Location	Severity	Imaging findings	TPI response	Surgery	Histopathology	Rehab
1	8 / M	1 month	Right inguinal	Mild-moderate	Not performed	Not performed	Neurotomy of ilioinguinal nerve	Not performed	None
2	12 / M	1 year	Left upper abdomen	Intermittent severe pain	Not performed	Temporary relief	Neurectomy	Vascular thrombosis	Yes (pre- and post-op)
3	22 / F	Several years	Left Upper abdomen	Intermittent severe pain	CT: Rectus abdominis atrophy, visible nerve	Temporary relief	Neurectomy	Perineural fibrosis	Yes (post-op)
4	12 / M	6 months	Left lower abdomen	Mild but disturbing	CT: Subtle nerve-like structure	Temporary relief	Neurectomy	Onion bulb-like structures	None
5	34 / M	2 years (post-COVID)	Right lower thorax	Electric shock-like pain	CT: Visible nerve, fascia thickening, muscle thinning	Temporary relief	Neurectomy, vascular ligation	Vacuolated axons	Yes (post-op)

Preoperative CT imaging was valuable in several patients, revealing abnormalities such as visible cutaneous nerves, fascial thickening, and localized rectus abdominis thinning. These findings not only supported diagnosis but also helped guide perioperative planning, including the need for rehabilitation. Notably, preoperative rehabilitation played a crucial role in reducing abdominal wall hypertonicity, facilitating precise localization of the most symptomatic nerve pathways. This effect has been similarly documented in previous conservative approaches to ACNES management, where targeted physical therapy alleviated muscle tension and improved nerve gliding mechanics, contributing to symptom relief [13]. Building upon the work of Newman et al., who demonstrated the effectiveness of a structured conservative rehabilitation approach for ACNES, our case series further supports the therapeutic value of perioperative rehabilitation [13]. In our experience, preoperative rehabilitation contributed not only to reducing abdominal wall hypertonicity but also to localizing the most symptomatic site, thereby enhancing surgical precision. Postoperative rehabilitation was equally valuable, promoting muscle improvement, relieving residual tension, and potentially preventing recurrence. These observations reinforce the importance of integrating rehabilitation into a comprehensive management plan for ACNES.

Although conservative treatment remains the first-line approach for ACNES, including nerve blocks and targeted rehabilitation, not all patients respond durably. In our series, we observed that some patients who reported only transient relief from nerve blocks and persistent symptoms despite rehabilitation were found to have histopathological changes such as perineural fibrosis, wavy nerve fibers, and vascular congestion upon surgical excision. These findings suggest the presence of irreversible nerve injury, which may underlie the refractory nature of their symptoms.

Our histopathological findings are consistent with early observations by Applegate et al., who demonstrated that the anterior cutaneous branches of the intercostal nerves traverse fibrous channels in the abdominal wall that can predispose them to entrapment [14]. The failure of conservative measures in these cases may reflect a point beyond which neural damage has become permanent, rendering further non-operative management ineffective. In such scenarios, surgical intervention should be considered promptly to prevent chronicity and deterioration of quality of life.

This aligns with the stepwise treatment algorithm proposed in previous literature, which advocates neurectomy as a reasonable next step for patients unresponsive to conservative therapy [2,5,6]. Our findings support this approach and suggest that histopathological changes could serve as a future basis for developing surgical indications in ACNES management.

Interestingly, one of our cases (case four) involved a patient who presented with relatively mild symptoms and no spontaneous pain during clinical visits. Nevertheless, due to repeated recurrence following nerve block injections, surgical intervention was ultimately pursued. Histopathological evaluation of the excised nerve revealed irreversible neuropathic changes, including perineural fibrosis and axonal distortion. This case underscores that the severity of clinical symptoms alone may not be a reliable criterion for determining surgical indication. Instead, a combination of clinical course, response to conservative treatment, and, when available in the future, histopathological insight should inform decision-making.

In case five, the patient presented with electric shock-like pain following a COVID-19 infection, and histopathological analysis revealed widespread nerve damage beyond a single segment, including axonal degeneration and perineural fibrosis. These findings suggest the possibility of a more diffuse neuropathic process rather than isolated mechanical entrapment. Given that SARS-CoV-2 has been associated with various peripheral neuropathies, including small fiber neuropathy and mononeuritis multiplex, post-infectious neuritis may have contributed to the pathogenesis in this case [15].

Moreover, intraoperative identification of a thick blood vessel adherent to the nerve implies that vascular proximity may further irritate the already vulnerable nerve fibers, potentiating chronic pain. The extent of the pathological changes indicates that conventional superficial neurectomy may be insufficient when neural involvement extends proximally. This aligns with recent suggestions that failed initial neurectomy may reflect an underestimation of the spatial distribution of neural injury [16].

Small fiber neuropathy, a known sequela of viral infection, typically presents with burning or electric pain and may lack overt motor findings. While it usually affects the distal limbs, rare reports have suggested that post-COVID small fiber neuropathy (SFN) may contribute to atypical presentations, including non-dermatomal truncal pain. However, current evidence remains limited. Diagnosis of SFN requires skin biopsy to assess intraepidermal nerve fiber density, and in the present case, no such test was performed. Should the patient experience recurrent or diffuse symptoms in the future, skin biopsy may help clarify the presence of underlying SFN [15, 17-19].

Importantly, this patient experienced complete and sustained resolution of symptoms following resection of both Th8 and Th9 anterior cutaneous nerves and ligation of the adjacent vessel. These results reinforce the need to consider post-viral neuropathy in ACNES-like presentations, especially when imaging and pathology suggest more widespread involvement. Deep-level or multi-segment neurectomy may be necessary in such contexts.

Particularly, case five presents a unique long COVID ACNES scenario, raising the possibility that viral inflammation or post-infectious changes may predispose patients to peripheral nerve entrapment. While the exact mechanism remains speculative, it highlights the need to consider ACNES in patients with persistent abdominal pain after COVID-19.

In summary, this case series emphasizes the importance of a comprehensive, individualized approach to the diagnosis and treatment of ACNES. While conservative therapies remain the first-line strategy, histopathological findings in our patients reveal that irreversible nerve damage can occur even in cases with mild symptoms or transient relief after nerve blocks. Preoperative imaging and rehabilitation not only aid in identifying the most symptomatic nerve segments but also optimize surgical outcomes. In cases of atypical or persistent pain, including those potentially triggered by post-viral mechanisms such as COVID-19, early consideration of surgical intervention may be warranted. These findings highlight the clinical utility of integrating anatomical, pathological, and functional assessments into a tailored treatment strategy for ACNES.

## Conclusions

This case series demonstrates that ACNES can present across a broad age range and may be misdiagnosed without careful clinical assessment. While conservative treatments, including nerve blocks and rehabilitation, remain the initial therapeutic strategy, their limited and transient efficacy in some cases highlights the necessity for timely surgical intervention. Histopathological analysis revealed evidence of irreversible nerve damage even in patients with mild or atypical symptoms, suggesting that symptom severity alone may not reliably guide surgical indication. Preoperative imaging and targeted rehabilitation not only improved diagnostic accuracy but also enhanced surgical outcomes. Furthermore, in cases with post-infectious neuropathy, such as following COVID-19, deeper and multi-segmental nerve involvement should be suspected. These findings underscore the value of a multidisciplinary, individualized treatment algorithm that integrates anatomical, pathological, and functional assessments to optimize outcomes for patients with ACNES.
